# Elevated serum insulin-like growth factor (IGF)-II and IGF binding protein-2 in patients with colorectal cancer

**DOI:** 10.1054/bjoc.2000.1462

**Published:** 2000-10-26

**Authors:** A G Renehan, J Jones, C S Potten, S M Shalet, S T O'Dwyer

**Affiliations:** Department of Surgery, Christie Hospital NHS Trust, Manchester, M20 4BX; Diabetic and Metabolic Laboratory, King's College School of Medicine and Dentistry, London; Cancer Research Campaign Department of Epithelial Biology, Paterson Institute for Cancer Research, Christie Hospital NHS Trust, Manchester; Department of Medicine and Endocrinology, Christie Hospital NHS Trust, Manchester, UK

**Keywords:** IGF-II, binding proteins, colorectal cancer

## Abstract

This study explored the relationships of serum insulin-like growth factors, IGF-I and IGF-II, and their binding proteins (IGFBP)-2 and IGFBP-3, with key clinicopathological parameters in 92 patients with colorectal cancer (cases). Comparisons were made with 57 individuals who had a normal colonoscopy (controls). Serial changes were examined in 27 cases. As IGF-related peptides are age- and sex-dependent, absolute concentrations were converted to standard deviation scores (SDS). Mean IGF-II SDS were elevated in Dukes A (*n*= 12 *P*< 0.001) and Dukes B (*n*= 25 *P*< 0.001) cases compared with controls, but not in advanced disease. Compared with controls, mean IGFBP-2 SDS were significantly elevated in patients with Dukes B (*P*< 0.001), Dukes C (*n*= 13 *P*< 0.001) and advanced disease (*n*= 42 *P*< 0.0001), with a significant trend from early to advanced disease (one-way ANOVA *P*< 0.001). Furthermore, IGFBP-2 SDS were positively related to tumour size (*P*= 0.01) and fell significantly in patients following curative resection (*P*= 0.04), suggesting that circulating levels reflect tumour load. We tested the potential tumour marker characteristics of IGFBP-2 SDS against three endpoints: metastasis alone; local pelvic recurrence alone; and metastasis and recurrence combined. The sensitivities for IGFBP-2 alone (≥ + 2SD) were modest at 55%, 46%, and 52%, but in combination with CEA, increased substantially to 90%, 77% and 86%, respectively. We conclude that the serum IGF-II and IGFBP-2 profiles may provide insights into underlying biological mechanisms, and that serum IGFBP-2 may have an adjunct role in cancer surveillance in patients with colorectal cancer. © 2000 Cancer Research Campaign


*British Journal of Cancer* (2000) **83**(10), 1344–1350
© 2000 Cancer Research Campaign
doi: 10.1054/ bjoc.2000.1462, available online at http://www.idealibrary.com on IDEAL®

### Elevated serum insulin-like growth factor (IGF)-II and IGF binding protein-2 in patients with colorectal cancer

**AG Renehan[1], J Jones[2], CS Potten[3], SM Shalet[4] and ST O'Dwyer[1]**

[1]Department of Surgery, Christie Hospital NHS Trust, Manchester M20 4BX; [2]Diabetic and Metabolic Laboratory, King's College School of Medicine and Dentistry, London; [3]Cancer Research Campaign Department of Epithelial Biology, Paterson Institute for Cancer Research, Christie Hospital NHS Trust, Manchester; [4]Department of Medicine and Endocrinology, Christie Hospital NHS Trust, Manchester, UK

**Summary** This study explored the relationships of serum insulin-like growth factors, IGF-I and IGF-II, and their binding proteins (IGFBP)-2 and IGFBP-3, with key clinicopathological parameters in 92 patients with colorectal cancer (cases). Comparisons were made with 57 individuals who had a normal colonoscopy (controls). Serial changes were examined in 27 cases. As IGF-related peptides are age- and sex-dependent, absolute concentrations were converted to standard deviation scores (SDS). Mean IGF-II SDS were elevated in Dukes A ($n = 12$, $P < 0.001$) and Dukes B ($n = 25$, $P < 0.001$) cases compared with controls, but not in advanced disease. Compared with controls, mean IGFBP-2 SDS were significantly elevated in patients with Dukes B ($P < 0.001$), Dukes C ($n = 13$, $P < 0.001$) and advanced disease ($n = 42$, $P < 0.0001$), with a significant trend from early to advanced disease (one-way ANOVA, $P < 0.001$). Furthermore, IGFBP-2 SDS were positively related to tumour size ($P = 0.01$) and fell significantly in patients following curative resection ($P = 0.04$), suggesting that circulating levels reflect tumour load. We tested the potential tumour marker characteristics of IGFBP-2 SDS against three endpoints: metastasis alone; local pelvic recurrence alone; and metastasis and recurrence combined. The sensitivities for IGFBP-2 alone (≥ + 2SD) were modest at 55%, 46%, and 52%, but in combination with CEA, increased substantially to 90%, 77% and 86%, respectively. We conclude that the serum IGF-II and IGFBP-2 profiles may provide insights into underlying biological mechanisms, and that serum IGFBP-2 may have an adjunct role in cancer surveillance in patients with colorectal cancer. © 2000 Cancer Research Campaign

**Keywords**: IGF-II; binding proteins; colorectal cancer

Insulin-like growth factor-I (IGF-I) and II (IGF-II) are regulatory peptides with a number of biological functions including cell proliferation, differentiation and anti-apoptosis (Jones and Clemmons, 1995; Le Roith, 1997). In the circulation, over 95% of IGF-I and IGF-II is bound to six high-affinity binding proteins (IGFBPs) (Rajaram et al, 1997). The major binding protein is IGFBP-3 which binds the IGF ligands forming a 150-kDa ternary complex with ALS (acid labile subunit). IGFBP-2 is the second most abundant IGF binding protein, binding IGF-II with a greater affinity (four-fold) than IGF-I (Clemmons, 1997). Most circulating IGF-I and IGF-II is synthesized in the liver but other tissues, including epithelial cells, may also contribute (Rajaram et al, 1997). Increased expression of IGF ligands and binding proteins has been recognized in a variety of human tumours (Macaulay, 1992), and consequently the contribution to the circulation of these peptides from a site other than that from normal hepatic synthesis may become significant in neoplastic processes.

In colorectal cancer, increased expression of IGF-II and IGFBP-2 mRNA has been noted in a number of colonic cancer lines (Tricoli et al, 1986; Lambert et al, 1992; Singh et al, 1996) and also, more recently, in human colonic adenocarcinomas (Mishra et al, 1997; Freier et al, 1999). IGF-I and IGF-II act via the IGF-I receptor, which is functionally expressed by human colon cancer lines (Lahm et al, 1994; Adenis et al, 1995). IGF activity may be further modulated by local levels of IGFBPs (Jones and Clemmons, 1995), indicating a potential complexity of regulatory mechanisms. Therefore, the measurement of circulating IGFs and their binding proteins in patients with cancer may not only reflect tumour presence but also provide insight into IGF-IGFBP inter-relationships at a cellular level.

We have previously reported that serum IGF-II levels are significantly elevated in healthy individuals (aged 55–64 years) with adenomas, known precursors of malignancy, found at screening flexible sigmoidoscopy (Renehan et al, 2000*a*). Furthermore, in the same study, elevated serum IGFBP-2 levels were found in those individuals with large adenomas (≥ 1 cm), and both IGF-II and IGFBP-2 values normalized after adenoma removal, implicating these peptides as potential tumour markers. In the present study, we have extended this work to examine the relationship between serum IGF-II and IGFBP-2 in patients with colorectal cancer compared with a control population of individuals with normal colonoscopic findings. The relationship between serum IGF-I and IGFBP-3 and colorectal cancer was also investigated as there is evidence that circulating IGF-I is positively and IGFBP-3 inversely, and independently, associated with cancer risk for prostate (Chan et al, 1998), breast (Hankinson et al, 1998) and colorectal cancer (Ma et al, 1999).

*Received 27 April 2000*
*Revised 12 July 2000*
*Accepted 18 July 2000*

*Correspondence to*: ST O'Dwyer

### PATIENTS AND METHODS

### Patients

Serum samples were collected from 92 patients (79 primary, 13 recurrent, median age = 61 (range 25–93) years) with colorectal

**Table 1** Clinicopathological characteristics

| | Primary ($n$ = 79) | Recurrent ($n$ = 13) |
|---|---|---|
| Median age (years) | 68 (range 25–93) | 58 (range 43–78) |
| Sex | | |
| Male | 41 (52) | 7 |
| Female | 38 (48) | 6 |
| Staging | | |
| Dukes' A | 12 (15) | |
| Dukes' B | 25 (32) | |
| Dukes' C | 13 (16) | |
| Advanced | | |
| distant metastasis (Dukes D)[a] | 29 (37) | |
| local pelvic recurrences | | 13 |
| Nutritional status | | |
| Well-nourished | 54 (68) | 10 |
| Malnourished | 25 (32) | 3 |
| Degree of differention[b] | | |
| Well | 10 (20) | |
| Moderate | 37 (74) | |
| Poor | 3 (6) | |

Values in parentheses are ranges and percentages; [a]All patients with distant metastases had hepatic lesions, in addition, three patients had pulmonary and four had intra-abdominal lesions. [b]Tumour differentiation quoted for the 50 patients undergoing primary definitive resection cancer at the time of presentation. The clinicopathological characteristics of these patients are shown in Table 1. Patients were categorized into two clinical groups: 50 patients with Dukes A, B or C tumours who underwent a definitive surgical resection; and 42 patients with advanced disease characterized by distant metastases at presentation i.e. 'Dukes D' ($n$ = 29), or local pelvic recurrence ($n$ = 13). Additional serum samples were obtained in 27 patients; 6–8 weeks following definitive surgery in 15 patients, and at variable times (median 5 weeks) during tumour progression in a further 12 patients. As nutritional status is known to influence circulatory levels of the IGFs and their binding proteins (Thissen et al, 1994), cancer patients were also categorized by nutritional status using the following criteria. Malnutrition was defined when at least two of the following were present in an individual patient: malnourished by global subjective assessment; body mass index less than 20 kg $m^{-2}$; mean arm circumference < 27 cm in males or < 26 cm in females; or albumin < 33 mg $l^{-1}$ (modified from Hammerlid et al, 1998). The control group comprised serum samples obtained from 57 individuals (median age = 60 (range 29–87) years, 20 males, 37 females) with normal colonoscopic findings and no history of previous colorectal neoplasia. The study was approved by the Local Ethics Committee for South Manchester Health Authorities.

### Clinicopathological parameters

Tumour stage was determined both clinically and on pathological evaluation. Surgically resected specimens were staged in accordance with Dukes classification (Turnbull et al, 1967) and graded by the degree of differentiation (well, moderate, poor) in accordance with the WHO classification (Jass and Sobin, 1989). For primary tumours treated by curative resection, size was the maximum tumour diameter and the site was classified as right colon (proximal to splenic flexure), left colon (splenic flexure to rectosigmoid junction) and rectum. The presence and extent of advanced disease (all had at least hepatic metastases) was determined using computerized tomographic (CT) or magnetic resonance (MR) scanning. Pelvic recurrence was confirmed by a combination of CT or MR scanning and examination under anaesthesia with biopsy for histological diagnosis.

### Assays

IGF-I was measured, following acid–alcohol extraction, by an established radioimmunoassay (RIA) using a polyclonal rabbit antiserum (R557A) raised against purified human IGF-I (Taylor et al, 1990; Toogood et al, 1998). Serum IGF-II was determined using a commercially available immunoradiometric assay (IRMA) kit (DSL, Webster, Texas, USA). IGFBP-2 and IGFBP-3 were measured using an RIA and IRMA, respectively (DSL). All samples were determined blind to cancer status and stage, and were assayed in triplicate. The inter-assay coefficients of variation (CV) at low, medium and high analyte levels were less than 10% for all four assays, with intra-assay CVs less than 5%. The sensitivities for IGF-I, IGF-II, IGFBP-2, and IGFBP-3, were 14 μg $l^{-1}$, 15 μg $l^{-1}$, 5 μg $l^{-1}$ and 0.5 mg $l^{-1}$, respectively. Carcinoembryonic antigen (CEA) was measured using a two-site (sandwich) chemilumuninescence system (Chiron Diagnostics, Halstead, UK). The threshold definition for an elevated CEA level was 5 ng $ml^{-1}$, with an analytical sensitivity of 0.5 ng $ml^{-1}$.

### Statistical analysis

As IGF ligands and binding proteins are age- and sex-dependent (Rajaram et al, 1997), absolute concentrations were converted to standard deviation scores (SDS) (SDS = $(x - X)$/SD: $x$ = measured value, $X$ = mean of normal values for age and sex of an individual, SD = standard deviation). Normal reference means and standard deviations were generated from an in-house dataset of 295 healthy individuals using the above assays (see Appendix for details). Differences in mean SDS were compared using Students t-test for independent means, paired t-tests, and one-way ANOVA as appropriate. Correlations were described by Pearsons coefficient ($r$). Tests were two-sided and a $P$-value less than 0.05 was considered to indicate statistical significance using SPSS 9.0 (Superior Performing Software Systems, Chicago, USA) for computations.

### RESULTS

### Validation of controls

For the 57 individuals with a normal colonoscopy, IGFBP-3 was significantly correlated with IGF-I ($r$ = 0.38, $P$ = 0.005) and IGF-II ($r$ = 0.85, $P$ < 0.001), similar to correlations seen in the normal reference data (see Appendix). The absolute concentrations for serum IGF-I, IGF-II, IGFBP-2 and IGFBP-3 in the 57 controls fell within the 90% predictive intervals as defined by the age–sex regression equations for normals in 96%, 100%, 98%, and 95% of values, respectively.

### Cases vs controls

When examined for all 92 patients with colorectal cancer, mean IGF-II SDS were marginally elevated compared with normal colonoscopy controls (mean ± SEM = 0.46 ± 0.18 vs 0.01 ± 0.09, $P$ = 0.06). There was no significant trend in IGF-II SDS across the Dukes stages (one-way ANOVA) but when analysed separately, mean SDS were significantly elevated in Dukes A (1.43 ± 0.34,

© 2000 Cancer Research Campaign

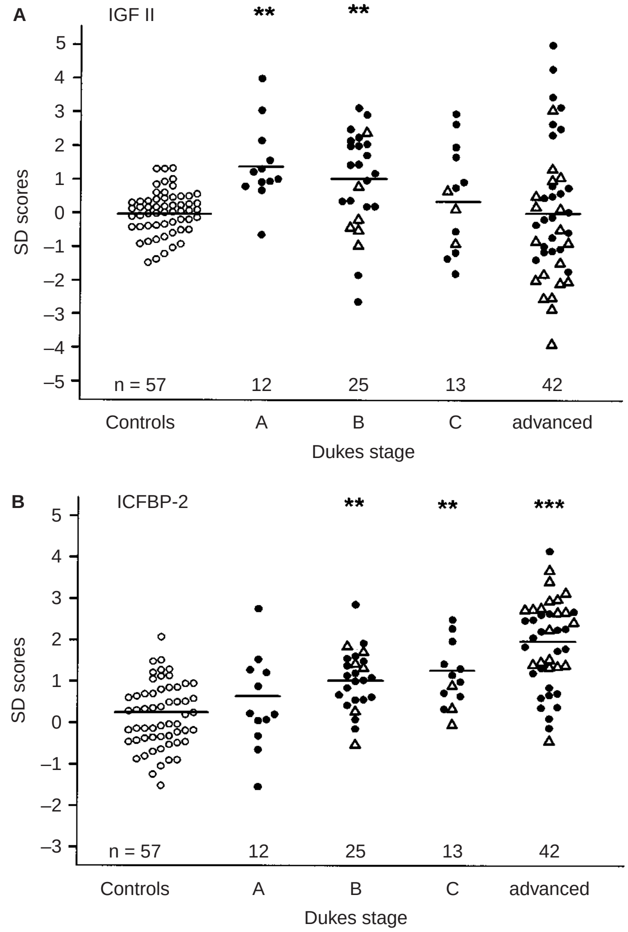

**Figure 1** IGF-II SDS (**A**) and IGFBP-2 SDS (**B**) shown for controls (normal colonoscopy) and stages in cancer patients (cases). Advanced disease include metastatic disease and local pelvic recurrences. Well-nourished cancer patients denoted ●; malnourished cancer patients Δ. Horizontal lines denote mean SDS for all patients in each subgroup; *$P$ < 0.001 compared with baseline controls; **$P$ < 0.0001 compared with baseline controls

**Table 2** IGF-I, IGF-II, IGFBP-2, and IGFBP-3 by nutritional status and disease status

| | SD scores (mean ± SEM) | | |
|---|---|---|---|
| | **Well-nourished** | **Malnourished**[a] | ***P* value**[b] |
| Number of patients | | | |
| early/advanced disease | 41/23 | 9/19 | – |
| IGF-I | | | |
| early (Dukes A, B, C) | 0.31 ± 0.20 | –0.66 ± 0.22 | 0.03 |
| advanced disease | –0.23 ± 0.29 | –1.00 ± 0.29 | 0.04 |
| IGF-II | | | |
| early (Dukes A, B, C) | 1.12 ± 0.23 | 0.11 ± 0.35 | 0.06 |
| advanced disease | 0.52 ± 0.36 | –0.86 ± 0.40 | 0.01 |
| IGFBP-2 | | | |
| early (Dukes A, B, C) | 0.96 ± 0.14 | 0.79 ± 0.28 | n.s. |
| advanced disease | 1.62 ± 0.22 | 2.21 ± 0.22 | 0.07 |
| IGFBP-3 | | | |
| early (Dukes A, B, C) | 1.36 ± 0.29 | 0.77 ± 0.41 | n.s. |
| advanced disease | 0.99 ± 0.35 | –0.20 ± 0.54 | 0.06 |

[a]See 'Patients and Methods' for criteria defining cancer-related malnutrition; [b]Student t-tests for independent means; n.s. = not significant

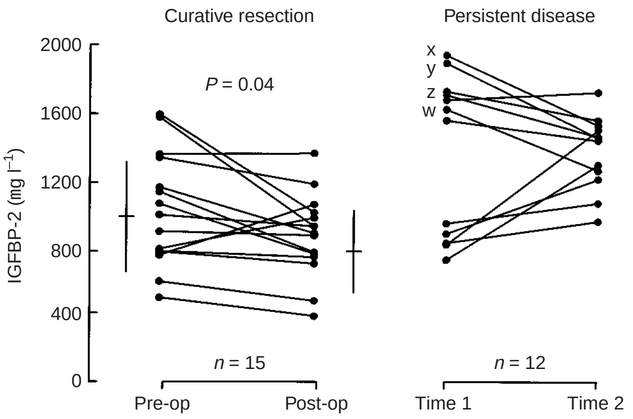

**Figure 2** Serial changes in serum IGFBP-2 concentrations in 15 patients undergoing curative resection and 12 patients with persistent disease. None of the 15 patients who had curative resection and a repeat blood sample was malnourished pre-surgery. Patients *x, y* and *z* received chemotherapy between time 1 and 2 with partial tumour response. Patient *w* had a rectal tumour debulked with argon ablation between time 1 and 2

$P$ < 0.001) and Dukes B (0.93 ± 0.29, $P$ < 0.001) patient groups (Figure 1A). A number of patients with Dukes C and advanced disease had greatly elevated IGF-II SDS but overall, the means were not raised. On the other hand, mean IGFBP-2 SDS was significantly raised in the total cancer patient group compared with controls (1.37 ± 0.12 vs 0.17 ± 0.10, $P$ < 0.0001). When considered by stage of disease, there was a significant trend toward increasing IGFBP-2 SDS from early to advanced disease (one-way ANOVA, $P$ < 0.0001), with significantly elevated means in Dukes B (1.03 ± 0.15, $P$ < 0.001), Dukes C (1.13 ± 0.22, $P$ < 0.001) and advanced disease (1.89 ± 0.16, $P$ < 0.0001) compared with controls (Figure 1B).

For all cases, there were no differences either in mean IGF-I SDS compared with controls (–0.19 ± 0.13 vs 0.17 ± 0.12, $P$ = 0.2) or mean IGFBP-3 SDS and controls (0.88 ± 0.20 vs 0.56 ± 0.14, $P$ = 0.6). When analysed by stage, there were no differences in mean IGF-I and IGFBP-3 SDS vs controls, with the exception that mean IGF-I SDS was significantly reduced in patients with advanced disease in whom malnutrition was prevalent (see below) (–0.59 ± 0.18 vs 0.17 ± 0.12, $P$ = 0.001).

### Effect of nutritional status

Malnutrition was present in six of 34 (18%) Dukes B patients, three of 13 (23%) Dukes C patients, and 19 of 42 (45%) patients with advanced disease. For all malnourished cancer patients, mean SDS were significantly reduced for IGF-I (malnourished vs controls = –0.87 ± 0.22 vs 0.19 ± 0.15, $P$ < 0.001), IGF-II (–0.48 ± 0.32 vs 1.04 ± 0.20, $P$ < 0.001), and IGFBP-3 (0.06 ± 0.42 vs 1.26 ± 0.23, $P$ = 0.04), but elevated for IGFBP-2 (1.68 ± 0.22 vs 1.16 ± 0.13, $P$ = 0.04). SDS values by nutritional status and disease status are shown in Table 2.

When the analysis was limited to well-nourished individuals, patterns of mean IGF-II and IGFBP-2 SDS for cases and controls were similar to the overall cohort. Of interest, however, after exclusion of malnutrition, mean IGF-II SDS were more

© 2000 Cancer Research Campaign

**Table 3** IGF-II and IGFBP-2 SD scores and various clinicopathological factors[a]

| | SD scores (mean ± SEM) | | | |
|---|---|---|---|---|
| | IGF-II | *P* value | IGFBP-2 | *P* value[b] |
| Tumour size[c] | | | | |
| < 3.5 cm ($n$ = 16) | 1.42 ± 0.41 | | 0.44 ± 0.23 | |
| 3.5–5.5 cm ($n$ = 18) | 1.08 ± 0.38 | | 0.87 ± 0.15 | |
| ≥ 5.5 cm ($n$ = 16) | 0.56 ± 0.39 | $P$ = n.s. | 1.33 ± 0.22 | $P$ = 0.01 |
| Differentiation | | | | |
| Well ($n$ = 11) | 1.34 ± 0.23 | | 0.79 ± 0.21 | |
| Moderate/poor ($n$ = 37/2) | 0.98 ± 0.25 | $P$ = n.s. | 0.85 ± 0.14 | $P$ = n.s. |
| Nodal status | | | | |
| No ($n$ = 37) | 1.09 ± 0.24 | | 0.80 ± 0.14 | |
| Yes ($n$ = 13) | 0.96 ± 0.44 | $P$ = n.s. | 0.93 ± 0.19 | $P$ = n.s. |
| Site distribution[d] | | | | |
| Right colon ($n$ = 9) | 1.13 ± 0.48 | | 0.85 ± 0.22 | |
| Left colon ($n$ = 15) | 1.22 ± 0.32 | | 0.79 ± 0.27 | |
| Rectum ($n$ = 26) | 0.94 ± 0.32 | $P$ = n.s. | 0.87 ± 0.15 | $P$ = n.s. |

[a]Based on the 50 patients undergoing primary definitive resection; [b]Student t-tests for independent means and one-way ANOVA, n.s. = not significant; [c]Tertiles of tumour diameter; [d]Right colon = proximal to splenic flexure; left colon = splenic flexure to rectosigmoid junction

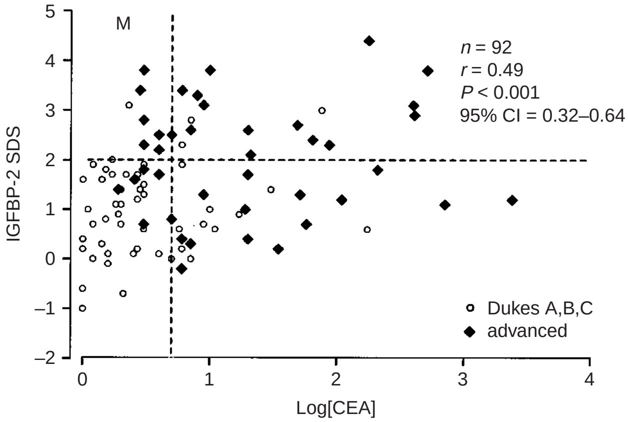

**Figure 3** Correlation between serum IGFBP-2 (SDS) and CEA concentrations ($\log_{10}$ transformed). Cut-off points for CEA (5 ng ml$^{-1}$ or $\log_{10}$[CEA] = 0.7) and IGFBP-2 (+2 SDS) shown as dotted lines. Of 26 patients with elevated IGFBP-2 SDS), seven had advanced disease but normal CEA values (quadrant marked M)

significantly elevated in patients with Dukes B (1.18 ± 0.34, $P < 0.001$), and also significantly elevated in Dukes C patients (0.63 ± 0.56, wide variance, $P = 0.05$) compared with colonoscopy controls.

### Relationship of IGF-II and IGFBP-2 and pathological characteristics

As both serum IGF-II and IGFBP-2 levels were elevated in cancer cases, we explored their relationships with a number of clinico-pathological characteristics among the 50 patients who underwent definitive surgical resection for Dukes A, B and C tumours (Table 3). There were no associations between IGF-II SDS and tumour size, differentiation, nodal status, and anatomic site, although there was a tendency for higher IGF-II SDS in small cancers compared with larger cancers. In contrast, IGFBP-2 SDS increased with increasing tumour size ($P = 0.01$), but similar to IGF-II showed no associations with degree of differentiation, nodal status or anatomic site.

**Table 4** Tumour marker characteristics of CEA and IGFBP-2

| | Sensitivity | Specificity | PPV[a] | NPV[b] |
|---|---|---|---|---|
| Metastases alone ($n$ = 29) | | | | |
| CEA (≥ 5 µg ml$^{-1}$) | 79% | 72% | 62% | 86% |
| IGFBP-2 (≥ + 2SD) | 55% | 92% | 80% | 78% |
| CEA and/or IGFBP-2 | 90% | 68% | 62% | 92% |
| Local pelvic recurrence alone ($n$ = 13) | | | | |
| CEA (≥ 5 µg ml$^{-1}$) | 62% | 71% | 36% | 88% |
| IGFBP-2 (≥ + 2SD) | 46% | 96% | 75% | 87% |
| CEA and/or IGFBP-2 | 77% | 69% | 40% | 92% |
| Combined metastases and recurrences ($n$ = 42) | | | | |
| CEA (≥ 5 µg ml$^{-1}$) | 72% | 74% | 69% | 77% |
| IGFBP-2 (≥ + 2SD) | 52% | 92% | 85% | 70% |
| CEA and/or IGFBP-2 | 86% | 68% | 69% | 85% |

[a]PPV = positive predictive value; [b]NPV = negative predictive value.

### Serial IGF-II and IGFBP-2 levels

We analysed serum IGF-II and IGFBP-2 values in 27 cancer patients before and after, or during treatments. In 15 patients undergoing curative resection for Dukes A, B and C tumours, there was a significant reduction in mean IGFBP-2 6–8 weeks following curative resection (mean ± SEM = 1011 ± 88 vs 860 ± 65 µg l$^{-1}$, paired t-test, $P = 0.04$), but no differences in mean IGF-II values. In a further 12 patients with persistent or progressive cancer, follow-up samples demonstrated elevated IGFBP-2 levels in eight (Figure 2).

### Tumour marker characteristics of IGFBP-2

We tested the potential tumour marker characteristics of IGFBP-2 SDS taking an arbitrary cut-off for elevated IGFBP-2 at ≥ +2 SDS. IGFBP-2 SDS were therefore elevated in 26 (28%) cases while serum CEA was elevated (≥ 5 ng ml$^{-1}$) in 42 (46%). IGFBP-2 SDS were significantly correlated with CEA ($r = 0.49$, $P < 0.001$), but of the 26 patients with elevated IGFBP-2 SDS, seven had advanced disease without elevated CEA values, suggesting that IGFBP-2 SDS may have independent predictive qualities (quadrant M in Figure 3). We therefore calculated sensitivities,

*© 2000 Cancer Research Campaign*

specificities, positive and negative predictive values for IGFBP-2 SDS alone, CEA alone, and both together, against three main endpoints: metastasis alone; local recurrence alone; and metastasis and recurrence combined (Table 4). By itself, the sensitivities for IGFBP-2 SDS were modest at 55%, 46% and 52%, respectively. In combination with CEA, however, the sensitivities for the three endpoints increased substantially to 90%, 77%, and 86%, respectively.

### DISCUSSION

This study has focused on the relationships of serum IGF-II and IGFBP-2 with colorectal cancer, and found that age–sex adjusted IGF-II values are significantly raised in patients with early cancers but seemingly not in advanced disease, and age–sex adjusted IGFBP-2 values increased significantly from early to advanced disease, and on average, were two standard deviations greater than controls in patients with metastatic and recurrent disease. Age–sex adjusted IGFBP-2 was also associated with tumour size and fell significantly in patients following curative tumour resection, suggesting that the circulating IGFBP-2 levels reflect tumour load. The sensitivities of serum IGFBP-2 alone as a marker of distant metastasis and/or recurrence were modest but increased substantially in combination with carcinoembryonic antigen (CEA). There were no associations between serum IGF-I and IGFBP-3, and the presence of cancer, but all four IGF-related peptides were significantly influenced by nutritional status.

Two small studies, from the same institute, have previously reported elevated IGF-II and IGFBP-2 levels in patients with colorectal cancer measured semi-quantitively from immunoblots (el Atiq et al, 1994; Baciuchka et al, 1998). In the present study, the use of radioimmunoassays and immunoradiometric assays afforded us the opportunity to investigate a large number of cases and controls, make adjustments for predicted age-and sex-related changes, and undertake subanalyses to evaluate the influences of different clinicopathological factors, nutritional status and treatment. Furthermore, the current study design carefully chose individuals with normal colonoscopic findings as controls, as serum IGF-II and IGFBP-2 levels may be elevated even in the presence of occult benign colorectal tumours (Renehan et al, 2000*a*).

The increase in serum IGF-II observed in patients with Dukes A and B colorectal cancers extends our observations that serum IGF-II is significantly raised in individuals with colorectal adenomas (Renehan et al, 2000*a*). At first glance, the lack of an IGF-II increase in patients with more advanced disease appears paradoxical. The paradox remained even after adjustment for nutritional status (malnutrition was prevalent among patients with more advanced disease and is a negative regulator of IGF-II) and suggests that there may be a down-regulation or post-transcriptional modification of IGF-II peptide expression with advancing disease. Consistent with the latter hypothesis, IGF-II immunohistochemical expression is absent in normal colonic epithelium, almost universally positive in adenomas (Renehan et al, 2000*a*), but present in only half of adenocarcinomas examined with high positivity scores limited to well differentiated cancers (observations from our laboratory). The relevance of raised serum IGF-II is unknown, but as IGF-II is both mitogenic and anti-apoptotic, it is generally perceived to be a factor favouring tumour progression (Macaulay, 1992). In support of this view, Kawamoto et al (1998) have observed that IGF-II immunopositivity predicts for poor prognosis in colorectal cancers.

The finding of raised serum IGFBP-2 in colorectal cancer patients is in accordance with other reports describing elevated IGFBP-2 levels in various malignancies including lung (Reeve et al, 1990), ovary (Kanety et al, 1996), prostate (Cohen et al, 1993; Ho and Baxter, 1997), Wilm's tumour (Zumkeller et al, 1993) and childhood leukaemia (Muller et al, 1994; Mohnike et al, 1996; Wex et al, 1998). In the absence of a clearly understood physiological role for IGFBP-2, these collective observations suggest that this binding protein may have a special role in malignancy. At a tissue level, through sequestration of ligand from its receptor, the effect of IGFBP-2 on the mitogenic action of IGF-I and IGF-II is generally considered inhibitory (Jones and Clemmons, 1995), and this has been shown to be the case in some IGF-responsive colonic carcinoma cell lines (Hoeflich et al, 1998). However, Hoeflich and colleagues (2000) have also reported a stimulatory effect of IGFBP-2 via IGF-I receptor-independent mechanisms in adenocortical tumour cells, and whether these pathways exist in colonic cancers is not yet known.

An alternative and attractive hypothesis for the role of increased IGFBP-2 in malignancy is that it serves as a storage pool for IGF-II (which binds with greater affinity than IGF-I) in the microenvironment of tumour cells. It has recently been recognized that an IGF-II/IGFBP-2 complex may partly bind to the extracellular matrix (ECM) (Arai et al, 1996) from where IGF-II may be liberated by proteolysis. A serine protease capable of degrading IGFBP-2 has been described, which leads to a reduction of the IGF binding capacity and liberation of its ligands into the pericellular environment (Gockerman and Clemmons, 1995). Based on this hypothetical model, the increased IGFBP-2 in the circulation may provide a reservoir for ECM-bound IGF/IGFBP-2 complexes in the vicinity of tumour cells, and thus the presence of elevated circulating IGFBP-2 could enhance tumour growth and progression.

We explored the characteristics of serum IGFBP-2 as a potential tumour marker and its relationship with serum CEA. The sensitivity of 72% for CEA detecting both distant metastases and recurrences combined is similar to values (66–85%) found in other studies (Wang et al, 1994; Wolf and Cohen 1997). Consistent with other studies (Moertel et al, 1993), we also found that the sensitivity of CEA to detect local pelvic recurrences was lower relative to its ability to detect distant metastases. Using a cut-off of +2 SDS, the sensitivities of IGFBP-2 alone for the detection of distant metastases and/or recurrences were modest but increased substantially when combined with CEA. This suggests a potential role for IGFBP-2 as an adjunct to CEA in monitoring patients with colorectal cancer. At a time when there is increasing evidence that intensive surveillance with early detection of recurrent and metastatic disease offers opportunities to improve survival (Renehan and O'Dwyer, 2000), prospective studies are now required to assess the benefits of serial IGFBP-2 monitoring (with CEA) in patients who have undergone curative treatment for colorectal cancer.

Whereas the characteristics of serum IGF-II and IGFBP-2 are best described as tumour markers, the characteristics of serum IGF-I and IGFBP-3 are best described as predictive for cancer risk. A number of recent epidemiological studies have demonstrated associations between circulating IGF-I and IGFBP-3 levels and cancer risk in various malignancies (Chan et al, 1998; Hankinson et al, 1998; Ma et al, 1999). Specifically for colorectal cancer, Ma and colleagues (1999) have reported that high–normal range IGF-I values and low–normal range IGFBP-3 values predict for subsequent cancer development. We have recently shown that the

*© 2000 Cancer Research Campaign*

same profile (high IGF-I/low IGFBP-3) predicts for 'high-risk' adenomas (Renehan et al, 2000*b*) but the current study was not specifically designed to assess cancer risk, as confounding factors such as altered nutritional status were expected (and subsequently demonstrated) in our cohort. For these reasons, we caution against drawing conclusions about the relationships of serum IGF-I, IGF-II and IGFBP-3 and colorectal cancer risk from studies using cross-sectional designs (Manousus et al, 1999).

This study, together with our previous observations in individuals with colorectal adenomas, have shown that there is a characteristic profile for serum IGF-II and IGFBP-2 from pre-malignant adenomas through early carcinomas to metastatic colorectal disease. We have speculated that these distinctive patterns may provide insight into underlying biological mechanisms. The potential of serum IGFBP-2 as a tumour marker in colorectal cancer has been demonstrated, but large prospective studies are required before definitive conclusions regarding its use in clinical practice can be drawn.

### ACKNOWLEDGEMENTS

We acknowledgement the permission of Mr E Kiff and Mr B Hancock for the inclusion of some of their patients in this study. We are indebted to the efforts of Dr A Toogood in compiling much of the normal reference dataset, and to the assistance of David Ryder in statistical analysis. This study was in part supported by Endocrine Endowment Funds and Surgical Research Funds, Christie Hospital NHS Trust. There are no conflicts of interest.

### REFERENCES

Adenis A, Peyrat JP, Hecquet B, Delobelle A, Depadt G, Quandalle P, Bonneterre J and Demaille A (1995) Type I insulin-like growth factor receptors in human colorectal cancer. *Eur J Cancer* **31a**: 50–55

Arai T, Busby W Jr and Clemmons DR (1996) Binding of insulin-like growth factor (IGF) I or II to IGF-binding protein-2 enables it to bind to heparin and extracellular matrix. *Endocrinology* **137**: 4571–4575

Baciuchka M, Remacle Bonnet M, Garrouste F, Favre R, Sastre B and Pommier G (1998) Insulin-like growth factor (IGF)-binding protein-3 (IGFBP-3) proteolysis in patients with colorectal cancer: possible association with the metastatic potential of the tumor. *Int J Cancer* **79**: 460–467

Chan JM, Stampfer MJ, Giovannucci E, Gann PH, Ma J, Wilkinson P, Hennekens CH and Polak M (1998) Plasma insulin-like growth factor-I and prostate cancer risk: a prospective study. *Science* **279**: 563–566

Clemmons DR (1997) Insulin-like growth factor binding proteins and their role in controlling IGF actions. *Cytokine Growth Factor Rev* **8**: 45–62

Cohen P, Peehl DM, Stamey TA, Wilson KF, Clemmons DR and Rosenfeld RG (1993) Elevated levels of insulin-like growth factor-binding protein-2 in the serum of prostate cancer patients. *J Clin Endocrinol Metab* **76**: 1031–1035

el Atiq F, Garrouste F, Remacle Bonnet M, Sastre B and Pommier G (1994) Alterations in serum levels of insulin-like growth factors and insulin-like growth-factor-binding proteins in patients with colorectal cancer. *Int J Cancer* **57**: 491–497

Freier S, Weiss O, Eran M, Flyvbjerg A, Dahan R, Nephesh I, Safra T, Shiloni E and Raz I (1999) Expression of the insulin-like growth factors and their receptors in adenocarcinoma of the colon. *Gut* **44**: 704–708

Gockerman A and Clemmons DR (1995) Porcine aortic smooth muscle cells secrete a serine protease for insulin-like growth factor binding protein-2. *Circ Res* **76**: 514–521

Hammerlid E, Wirblad B, Sandin C, Mercke C, Edstrom S, Kaasa S, Sullivan M and Westin T (1998) Malnutrition and food intake in relation to quality of life in head and neck cancer patients. *Head Neck* **20**: 540–548

Hankinson SE, Willett WC, Colditz GA, Hunter DJ, Michaud DS, Deroo B, Rosner B, Speizer FE and Pollak M (1998) Circulating concentrations of insulin-like growth factor-I and risk of breast cancer. *Lancet* **351**: 1393–1396

Ho PJ and Baxter RC (1997) Insulin-like growth factor-binding protein-2 in patients with prostate carcinoma and benign prostatic hyperplasia. *Clin Endocrinol (Oxf)* **46**: 333–342

Hoeflich A, Lahm H, Blum W, Kolb H and Wolf E (1998) Insulin-like growth factor-binding protein-2 inhibits proliferation of human embryonic kidney fibroblasts and of IGF-responsive colon carcinoma cell lines. *FEBS Lett* **434**: 329–334

Hoeflich A, Fettscher O, Lahm H, Kolb H, Wolf E, Engelhardt D and Weber MM (2000) Overexpression of insulin-like growth factor-binding protein-2 results in increased tumorogenic potential in Y-1 adrenocortical tumor cells. *Cancer Res* **60**: 834–838

Jass JR and Sobin LH (1989) *Histological typing of intestinal tumors, 2nd edn*, World Health Organisation: Geneva

Jones JI and Clemmons DR (1995) lnsulin-like growth factors and their binding proteins: biological actions. *Endocr Rev* **16**: 3–B4

Juul A, Main K, Blum WF, Lindholm J, Ranke MB and Skakkebaek NE (1994) The ratio between serum levels of insulin-like growth factor (IGF)-I and the IGF binding proteins (IGFBP-1, 2 and 3) decreases with age in healthy adults and is increased in acromegalic patients. *Clin Endocrinol (Oxf)* **41**: 85–93

Kanety H, Kattan M, Goldberg I, Kopolovic J, Ravia J, Menczer J and Karasik A (1996) Increased insulin-like growth factor binding protein-2 (IGFBP-2) gene expression and protein production lead to high IGFBP-2 content in malignant ovarian cyst fluid. *Br J Cancer* **73**: 1069–1073

Kawamoto K, Onodera H, Kondo S, Kan S, Ikeuchi D, Maetani S and Imamura M (1998) Expression of insulin-like growth factor-2 can predict the prognosis of human colorectal cancer patients: correlation with tumor progression, proliferative activity and survival. *Oncology* **55**: 242–248

Lahm H, Amstad P, Wyniger J, Yilmaz A, Fischer JR, Schreyer M and Givel JC (1994) Blockade of the insulin-like growth-factor-I receptor inhibits growth of human colorectal cancer cells: evidence of a functional IGF-II-mediated autocrine loop. *Int J Cancer* **58**: 452–459

Lambert S, Carlisi A, Collette J, Franchimont P and Gol Winkler R (1992) Insulin-like growth factor II in two human colon-carcinoma cell lines: gene structure and expression, and protein secretion. *Int J Cancer* **52**: 404–408

Le Roith D (1997) Seminars in medicine of the Beth Israel Deaconess Medical Center. Insulin-like growth factors. *N Engl J Med* **336**: 633–640

Ma J, Pollak MN, Giovannucci E, Chan JM, Tao Y, Hennekens CH and Stampfer MJ (1999) Prospective study of colorectal cancer risk in men and plasma levels of insulin-like growth factor (IGF)-I and IGF-binding protein-3. *J Natl Cancer Inst* **91**: 620–625

Macaulay VM (1992) Insulin-like growth factors and cancer. *Br J Cancer* **65**: 311–320

Manousos O, Souglakos J, Bosetti C, Tzonou A, Chatzidakis V, Trichopoulos D, Adami HO and Mantzoros C (1999) IGF-I and IGF-II in relation to colorectal cancer. *Int J Cancer* **83**: 15–17

Mishra L, Bass B, Ooi BS, Sidawy A and Korman L (1997) Role of insulin-like growth factor-I (IGF-I) receptor, IGF-I, and IGF binding protein-2 in human colorectal cancers. *Growth Regulation* **7**: 1–10

Moertel CG, Fleming TR, Macdonald JS, Haller DG, Laurie JA and Tangen C (1993) An evaluation of the carcinoembryonic antigen (CEA) test for monitoring patients with resected colon cancer. *JAMA* **270**: 943–947

Mohnike KL, Kluba U, Mittler U, Aumann V, Vorwerk P and Blum WF (1996) Serum levels of insulin-like growth factor-I, -II and insulin-like growth factor binding proteins -2 and -3 in children with acute lymphoblastic leukaemia. *Eur J Pediatr* **155**: 81–86

Muller HL, Oh Y, Lehrnbecher T, Blum WF and Rosenfeld RG (1994) Insulin-like growth factor-binding protein-2 concentrations in cerebrospinal fluid and serum of children with malignant solid tumors or acute leukemia. *J Clin Endocrinol Metab* **79**: 428–434

Rajaram S, Baylink DJ and Mohan S (1997) Insulin-like growth factor-binding proteins in serum and other biological fluids: regulation and functions. *Endocr Rev* **18**: 801–831

Reeve JG, Payne JA and Bleehen NM (1990) Production of immunoreactive insulin-like growth factor-I (IGF-I) and IGF-I binding proteins by human lung tumours. *Br J Cancer* **61**: 727–731

Renehan AG and O'Dwyer ST (2000) Surveillance after colorectal cancer resection. *Lancet* **355**: 1095–1096

Renehan AG, Painter JE, O'Halloran D, Atkin WS, Potten CS, O'Dwyer ST and Shalet SM (2000*a*) Circulating insulin-like growth factor (IGF)-II and colorectal adenomas. *J Clin Endocrinol Metab* **85**: 3402–3408

Renehan AG, Painter JE, Atkin WS, Potten CS, Shalet SM and O'Dwyer ST (2000*b*) 'high-risk' colorectal adenomas and serum insulin-like growth factors. *Br J Surg* (in press)

Singh P, Dai B, Yallampalli U, Lu X and Schroy PC (1996) Proliferation and differentiation of a human colon cancer cell line (CaCo2) is associated with

*© 2000 Cancer Research Campaign*

significant changes in the expression and secretion of insulin-like growth factor (IGF) IGF-II and IGF binding protein-4: role of IGF-II. *Endocrinology* **137**: 1764–1774
Taylor AM, Dunger DB, Preece MA, Holly JM, Smith CP, Wass JA, Patel S and Tate VE (1990) The growth hormone independent insulin-like growth factor-I binding protein BP-28 is associated with serum insulin-like growth factor-I inhibitory bioactivity in adolescent insulin-dependent diabetics. *Clin Endocrinol Oxf* **32**: 229–239
Thissen JP, Ketelslegers JM and Underwood LE (1994) Nutritional regulation of the insulin-like growth factors. *Endocr Rev* **15**: 80–101
Toogood AA, Jones J, PA O'Neill, Thorner MO and Shalet SM (1998) The diagnosis of severe growth hormone deficiency in elderly patients with hypothalamic-pituitary disease. *Clin Endocrinol Oxf* **48**: 569–576
Tricoli JV, Rall LB, Karakousis CP, Herrera L, Petrelli NJ, Bell GI and Shows TB (1986) Enhanced levels of insulin-like growth factor messenger RNA in human colon carcinomas and liposarcomas. *Cancer Res* **46**: 6169–6173
Turnbull R, Kyle K and Watson F (1967) Cancer of the colon: the influence of the no-touch technique on survival rates. *Ann Surg* **166**: 420–427
Wang JY, Tang R and Chiang JM (1994) Value of carcinoembryonic antigen in the management of colorectal cancer. *Dis Colon Rectum* **37**: 272–277
Wex H, Vorwerk P, Mohnike K, Bretschneider D, Kluba U, Aumann V, Blum WF and Mittler U (1998) Elevated serum levels of IGFBP-2 found in children suffering from acute leukaemia is accompanied by the occurrence of IGFBP-2 mRNA in the tumour clone. *Br J Cancer* **78**: 515–520
Wolf RF and Cohen AM (1997) The miniscule benefit of serial carcinoembryonic antigen monitoring after effective curative treatment for primary colorectal cancer. *J Am Coll Surg* **185**: 60–64
Yu H, Mistry J, Nicar MJ, Khosravi MJ, Diamandis A, van Doorn J and Juul A (1999) Insulin-like growth factors (IGF-I, Free IGF-I, and IGF-II) and insulin-like growth factor binding proteins (IGFBP-2, IGFBP-3, IGFBP-6, and ALS) in blood circulation. *J Clin Lab Analysis* **13**: 166–172
Zumkeller W, Schwander J, Mitchell CD, Morrell DJ, Schofield PN and Preece MA (1993) Insulin-like growth factor (IGF)-I, -II and IGF binding protein-2 (IGFBP-2) in the plasma of children with Wilms' tumour. *Eur J Cancer* **29a**: 1973–1977

### APPENDIX

Age- and sex-normal reference means, standard deviations and 90% predictive ranges for IGF-I, IGF-II, IGFBP-2 and IGFBP-3 were calculated from regression plots using SigmaPlot 2.0 (Jandel Scientific Graphing Software, Erkrath, Germany) from measurements in 295 healthy individuals (162 males, 133 females, age 20–90 years). Mean IGF-I, IGF-II and IGFBP-3 levels declined steadily with age, whereas mean IGFBP-2 levels increased. IGFBP-3 was strongly correlated with IGF-I ($r$ = 0.77, $P$ < 0.001) and IGF-II ($r$ = 0.76, $P$ < 0.001), while IGFBP-2 was negatively correlated with IGF-I ($r$ = –0.33, $P$ < 0.001) and IGF-II ($r$ = –0.36, $P$ < 0.001). Mean IGF-I levels tended to be lower in females compared with males, while the reverse was seen for IGF-II and IGFBP-3. The sex differences for IGFBP-2 were small. These relationships were best described mathematically by second-order regression equations (below). The age- and sex-related changes in our normal reference dataset are similar to those described in other surveys (Juul et al, 1994; Yu et al, 1999).

| | Unit | Regression equations | SD |
|---|---|---|---|
| IGF-I | | | |
| Males | µg l$^{-1}$ | y = (0.0500x – 7.7835)x + 453.9 | 57 |
| Females | µg l$^{-1}$ | y = (0.0478x – 8.2959)x + 471.9 | 54 |
| IGF-II | | | |
| Males | µg l$^{-1}$ | y = (–0.0717x + 0.7326)x + 904.3 | 135 |
| Females | µg l$^{-1}$ | y = (–0.0300x – 1.0790)x + 921.3 | 155 |
| IGFBP-2 | | | |
| Males | µg l$^{-1}$ | y = (0.2920x – 23.667)x + 926.8 | 373 |
| Females | µg l$^{-1}$ | y = (0.2441x – 15.495)x + 696.1 | 398 |
| IGFBP-3 | | | |
| Males | mg l$^{-1}$ | y = (0.0005x – 0.0823)x + 5.8245 | 0.38 |
| Females | mg l$^{-1}$ | y = (0.0005x – 0.0812)x + 5.8245 | 0.48 |

© 2000 Cancer Research Campaign
